# Short chain ceramides disrupt immunoreceptor signaling by inhibiting segregation of Lo from Ld Plasma membrane components

**DOI:** 10.1242/bio.034702

**Published:** 2018-08-10

**Authors:** David Holowka, Kankanit Thanapuasuwan, Barbara Baird

**Affiliations:** Department of Chemistry and Chemical Biology, Cornell University, Ithaca, New York 14853, USA

**Keywords:** Immunoreceptor signaling, Lipid rafts, Phase segregation

## Abstract

Lipid phase heterogeneity in plasma membranes is thought to play a key role in targeting cellular signaling, but efforts to test lipid raft and related hypotheses are limited by the spatially dynamic nature of these phase-based structures in cells and by experimental characterization tools. We suggest that perturbation of plasma membrane structure by lipid derivatives offers a general method for assessing functional roles for ordered lipid regions in membrane and cell biology. We previously reported that short chain ceramides with either C2 or C6 acyl chains inhibit antigen-stimulated Ca^2+^ mobilization (Gidwani et al., 2003). We now show that these short chain ceramides inhibit liquid order (Lo)-liquid disorder (Ld) phase separation in giant plasma membrane vesicles that normally occurs at low temperatures. Furthermore, they are effective inhibitors of tyrosine phosphorylation stimulated by antigen, as well as store-operated Ca^2+^ entry. In Jurkat T cells, C6-ceramide is also effective at inhibiting Ca^2+^ mobilization stimulated by either anti-TCR or thapsigargin, consistent with the view that these short chain ceramides effectively interfere with functional responses that depend on ordered lipid regions in the plasma membrane.

## Abstract (precis)

**Summary:** Our manuscript describes how perturbation of plasma membrane structure by short chain ceramides offers a general method for assessing functional roles for ordered lipid regions in membrane and cell biology.

## INTRODUCTION

Although the biological relevance of lipid phase-like properties in the plasma membrane, including ordered lipid domains (‘lipid rafts’), has been controversial (Munro, 2003), strong evidence supports the general consensus that such lipid-based heterogeneity participates in many aspects of cell biology involving the plasma membrane (for recent review see Sezgin et al., 2017). A general method to disrupt membrane lipid order would be useful for evaluating its functional roles. Using a wide range of experimental approaches in a mast cell model system, we established a consistent mechanism by which antigen-crosslinked IgE/FcεRI complexes associate with liquid order (Lo)-preferring lipids, thereby stabilizing raft-like structures that preferentially include membrane-anchored Lyn tyrosine kinase and exclude transmembrane tyrosine phosphatases (Holowka et al., 2005). This stimulated co-segregation results in tyrosine phosphorylation of FcεRI subunits to initiate the signaling cascade and consequent activation of store-operated Ca^2+^ entry (SOCE), which leads to exocytosis and other manifestations of mast cell responses to the antigen stimulus (Holowka et al., 2012).

**Fig. 1.**
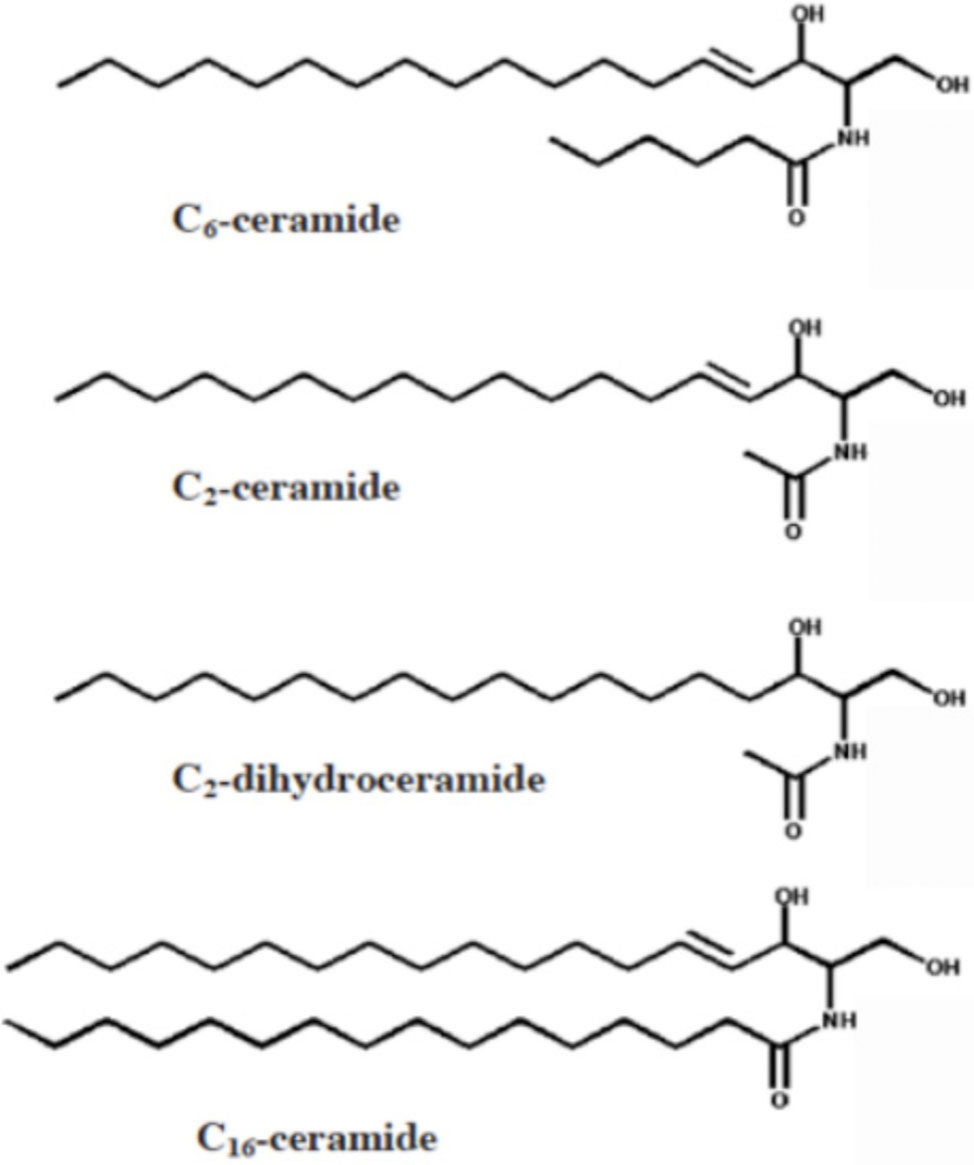
**Structures of short chain ceramides evaluated in this study.** N-hexanoyl-D-sphingosine (C6-ceramide), N-acetyl-D-sphingosine (C2-ceramide) and C2-dihydroceramide. N-palmitoyl-D-sphingosine (C16-ceramide) is a natural hydrolysis product of sphingomyelin by endogenous sphingomyelinases. Reproduced from Gidwani et al. (2003).

In a previous study we found that depletion of cholesterol, a key component of Lo-like phases, inhibits the earliest signaling events in FcεRI signaling (Sheets et al., 1999). Sphingomyelin is also known to be a major component of these ordered lipid domains (Pike, 2006). Ceramides are the products of sphingomyelin hydrolysis by sphingomyelinases, which cleave off the choline head group to yield long-chain neutral lipids with a sphingosine backbone and a long chain fatty acid in an amide linkage (e.g. C16-ceramide, Fig. 1). Ceramides with short acyl chains (e.g. C2- and C6-ceramide, Fig. 1), which can be added exogenously and redistribute readily across membranes, have been used to mimic their long acyl chain counterparts. When sphingomyelinases and short chain ceramides are compared, many cell biological effects are similar, but some are different. C2-dihydroceramide (Fig. 1) was found to have little biological activity and is often used as a control (Simon and Gear, 1998; Kolesnick et al., 2000; Hannun and Obeid, 2002). Chiantia et al. (2007) used atomic force microscopy (AFM) and fluorescence correlation spectroscopy (FCS) to investigate the effect of ceramide chain length on the phase properties of model membranes. They found that, unlike long-chain ceramides (e.g. C18 and C16), which segregate from the liquid disorder (Ld) phase, short-chain ceramides perturb the lipid packing of the Lo domains. For example, they found that C2- and C6-ceramide effectively increase the lateral diffusion coefficient of a fluorescent cholesterol derivative, bodipy-cholesterol, in the Lo phase, but do not significantly alter its diffusion in the Ld phase.

We previously showed that C2- and C6- short chain ceramides decrease the degree of lipid order, as measured by fluorescence anisotropy, in plasma membrane vesicles derived from rat basophilic leukemia (RBL) mast cells, and increase the distance between Lo-preferring proteins, as measured by fluorescence resonance energy transfer (FRET) in intact cells. In contrast, these effects were not seen with C2-dihydroceramide. Parallel experiments in that study showed C2- and C6-ceramides (but not C2-dihydroceramide) to be effective inhibitors of stimulated Ca^2+^ mobilization in these cells (Gidwani et al., 2003). This cellular response was stimulated by crosslinking of IgE bound to its high affinity receptor, FcεRI, by a multivalent antigen that specifically binds to the IgE combining sites. Although our earlier experiments designed to evaluate the specific mechanism of inhibition were inconclusive, we observed that the first step in Ca^2+^ mobilization, IP_3_-dependent release of Ca^2+^ from ER stores, is sensitive to inhibition by these short chain ceramides.

As described herein, we investigated whether these short chain ceramides serve as a more general inhibitor of ordered lipid domains, specifically interfering with the initiating steps in this signaling cascade, particularly Lyn-mediated tyrosine phosphorylation stimulated by antigen-crosslinked IgE-FcεRI. We monitored the kinetics of inhibition of antigen- and thapsigargin-stimulated Ca^2+^ mobilization. To test the generality of these observations, we further evaluated inhibition by short chain ceramides of signaling in related responses to the T cell receptor for antigen.

## RESULTS

**Fig. 2.**
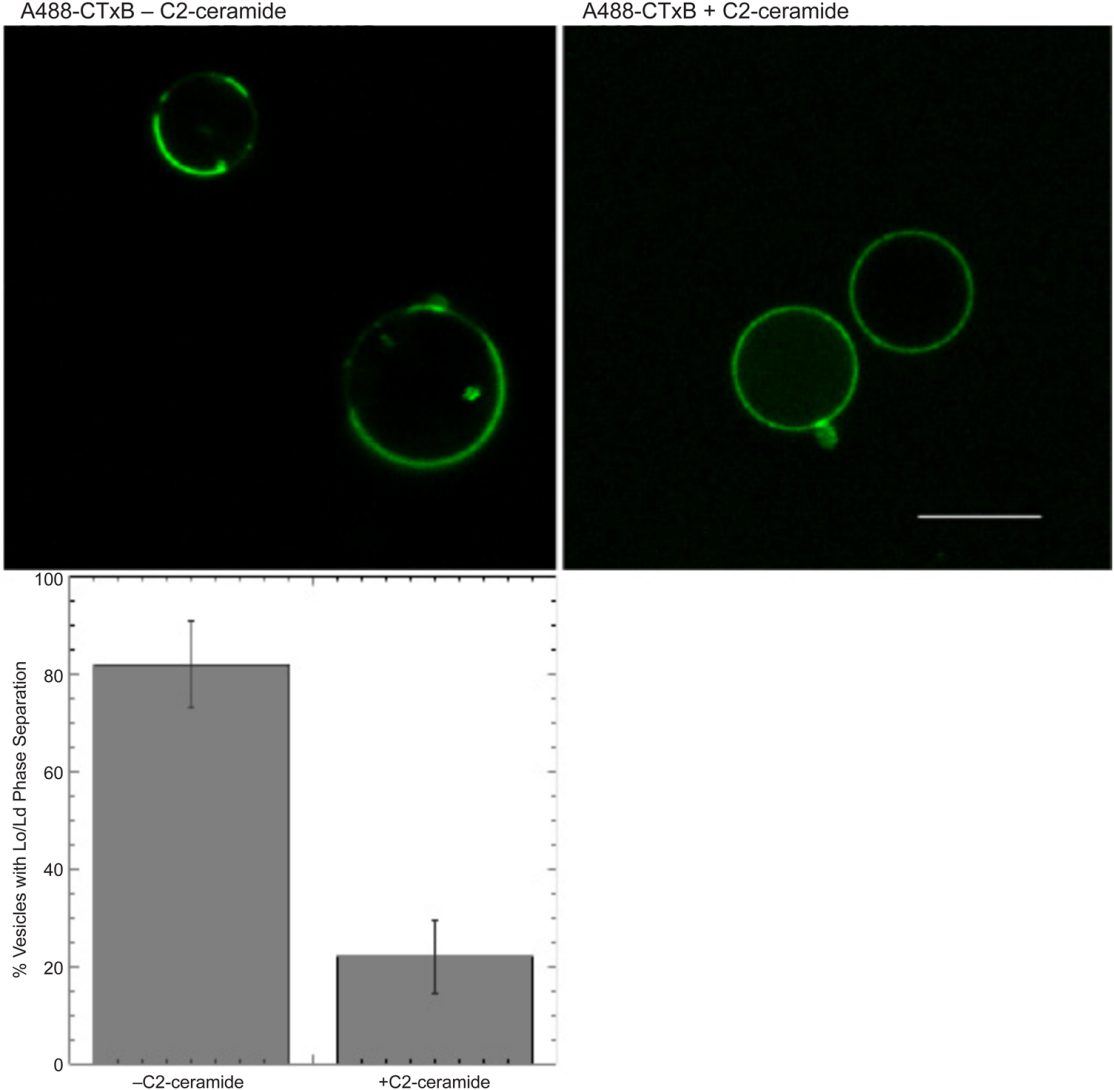
**C2-ceramide inhibits Lo/Ld phase separation in giant plasma membrane vesicles.** Top panels: confocal images of GPMVs from RBL cells labeled with A488-cholera toxin B (an Lo marker) and cooled on ice in the absence (left) or presence (right) of 32 μM C2-ceramide. Scale bar: 10 μm. Lower panel: summary of scoring >180 GPMVs for each sample by fluorescence imaging in three separate experiments (±s.d.).

Because our previous results suggested that the short chain ceramides, C2- and C6- (Fig. 1), inhibit Ca^2+^ mobilization by interfering with ordered lipid membrane structure, we examined their effects on the Lo/Ld-like phase separation that occurs in giant plasma membrane vesicles (GPMVs) derived from RBL mast cells following treatment to induce their formation and detachment from the plasma membrane skeleton (Baumgart et al., 2007). As represented in Fig. 2, top left panel, GPMVs labeled with an Lo marker, AlexaFluor488-cholera toxin B (A488-CTXB) and cooled below ambient temperature show large-scale phase separation of the labeled Lo phase from the unlabeled Ld phase in a large percentage of vesicles. Labeled GPMVs that were pretreated with an optimal dose of 32 μM C2-ceramide prior to cooling exhibit uniform labeling under these conditions (Fig. 2, top right panel). As summarized for three separate experiments in the lower panel of Fig. 2, ∼80% of the GPMVs phase separated in the absence of C2-ceramide under these conditions, whereas only ∼20% phase separated in the presence of C2-ceramide. Other experiments provided evidence that C2-ceramide reduces the average fluidity of the plasma membrane outer leaflet in live cells using the lipid probe tetramethylammonium-diphenylhexatriene and monitoring its steady-state fluorescence anisotropy, suggesting that C2-ceramide interferes with Lo lipid packing (K.T., D.H., unpublished) as shown previously for model membranes (Chiantia et al., 2007). Together, these results indicate that C2-ceramide inhibits plasma membrane Lo/Ld-like phase separation, suggesting a plausible basis for its inhibition of stimulated Ca^2+^ mobilization in these cells.

**Fig. 3.**
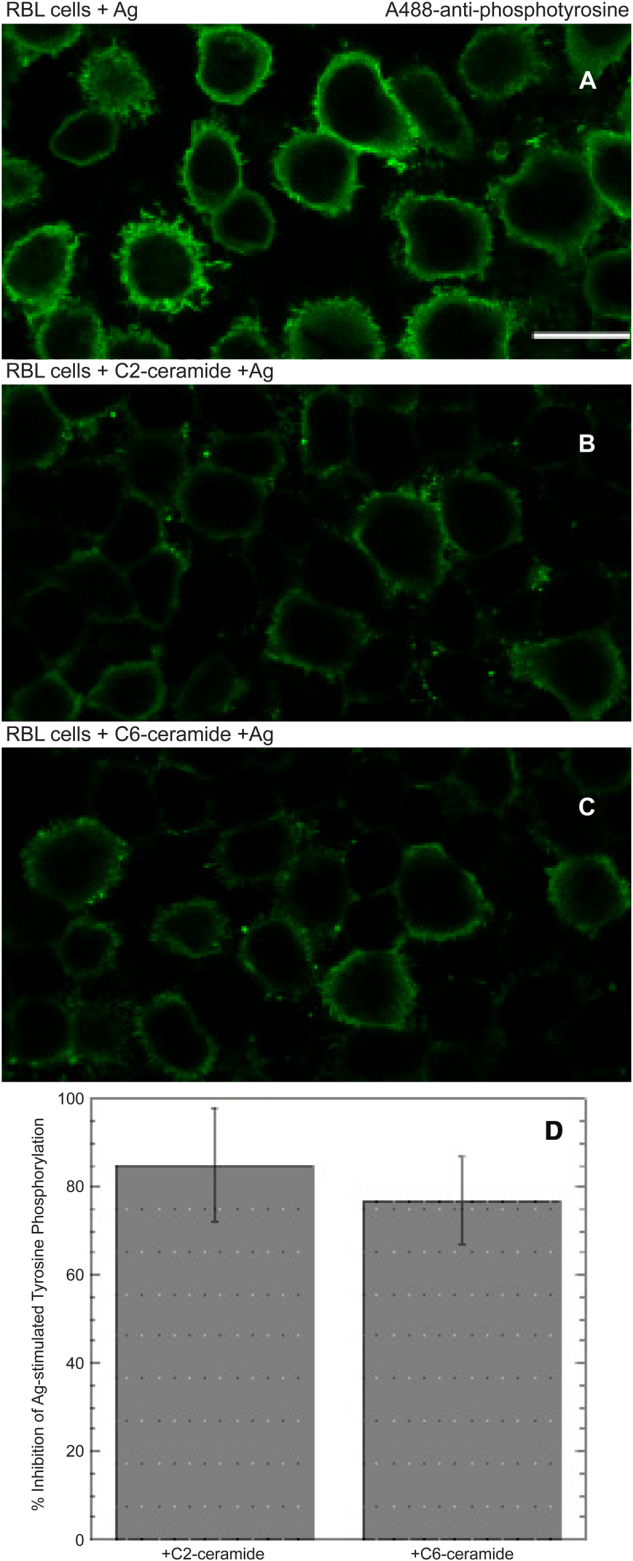
**C2-ceramide and C6-ceramide inhibit antigen-stimulated tyrosine phosphorylation in RBL mast cells.** Anti-DNP IgE-sensitized cells were stimulated for 5 min at 37°C with an optimal dose of DNP_16_-BSA in the absence (A) or the presence of C2-ceramide (B) or C6-ceramide (C), then fixed, permeabilized, and labeled with mAb 4G10 anti-phosphotyrosine+A488-labeled with 2^o^ Ab. Identical optical setting were used for all three panels. Scale bar: 10 μm. (D) Summary of quantitation from three experiments with >250 cells for each condition, ±s.d.

We previously showed that the first step in Ca^2+^ mobilization by antigen-stimulated release of Ca^2+^ from ER stores is sensitive to inhibition by short chain ceramides (Gidwani et al., 2003). We investigated whether these ceramide derivatives inhibit an earlier step in FcεRI signaling: tyrosine phosphorylation that is stimulated by crosslinking of IgE bound to FcεRI. To assess this process, we utilized the monoclonal anti-phosphotyrosine antibody 4G10 that is widely used to detect stimulated tyrosine phosphorylation in mammalian cells (Ley et al., 1991). RBL cells sensitized with anti-DNP IgE were stimulated with an optimal dose of multivalent DNP-BSA for 5 min at 37°C in the presence or absence of either 32 µM C2-ceramide or 16 µM C6-ceramide before cells were fixed, permeabilized, and labeled with 4G10. As represented in Fig. 3A-C, stimulated tyrosine phosphorylation is observed primarily at the plasma membrane, as expected, and this is severely reduced by preincubation with either C2- or C6-ceramide. As summarized in Fig. 3D for three separate experiments, this response is inhibited by ∼75-85% for C2-ceramide and C6-ceramide in these cells.

**Fig. 4.**
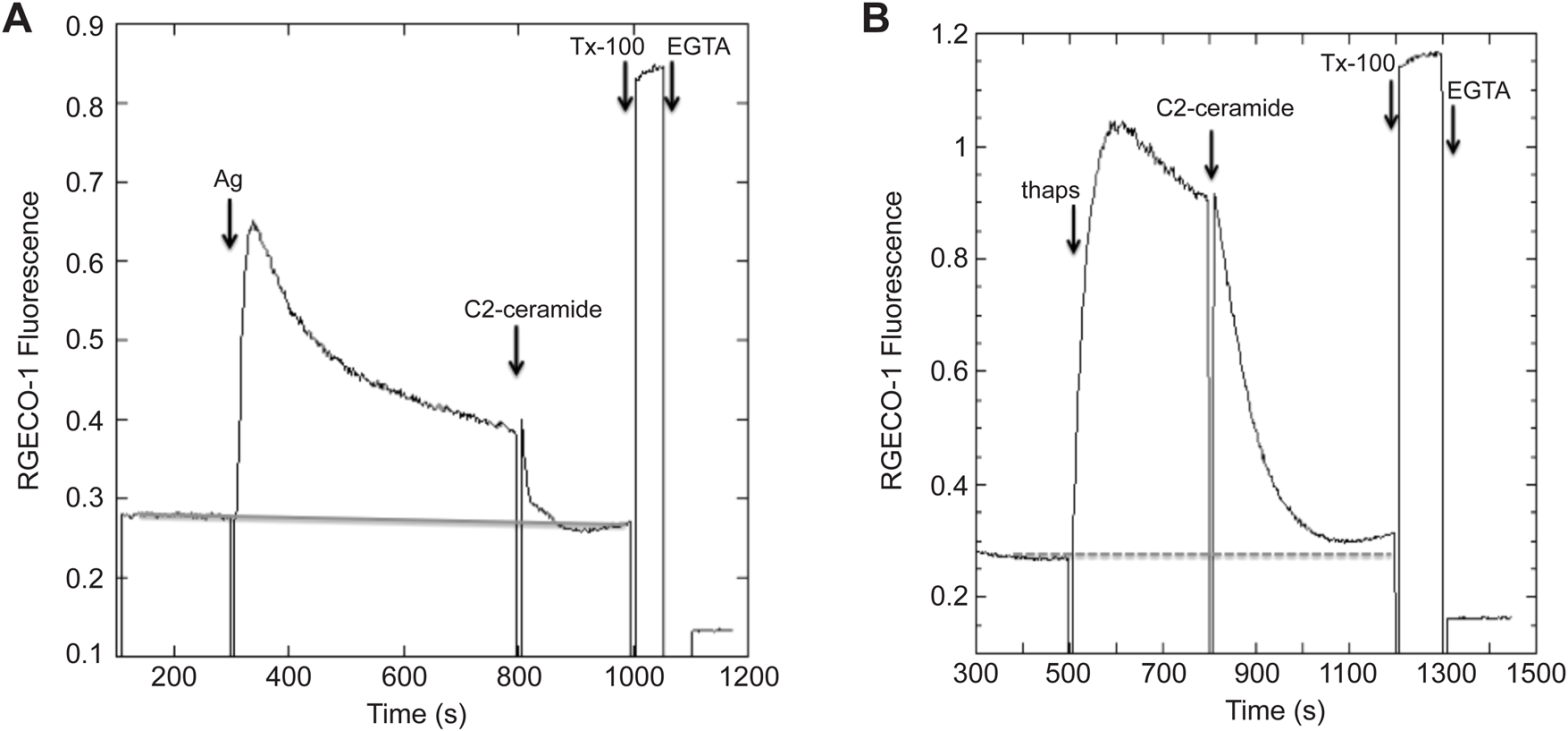
**Effects of C2-ceramide on stimulated calcium responses in RBL cells.** C2-ceramide, added acutely at a final concentration of 32 μM, effectively inhibits antigen-stimulated (A) and thapsigargin-stimulated (B) Ca^2+^ mobilization in RBL mast cells.

We previously characterized inhibition of antigen-stimulated Ca^2+^ responses in RBL cells that had been pre-equilibrated with these short chain ceramides (Gidwani et al., 2003). To assess the kinetics of this inhibition, we monitored the Ca^2+^ response following acute addition of these derivatives to cells, after they had been stimulated by antigen. As shown in Fig. 4A, this inhibition occurs with a half-time of ∼20 s with 32 µM C2-ceramide and an optimal dose of antigen. We also monitored inhibition of the Ca^2+^ response stimulated by thapsigargin, which bypasses the early steps of FcεRI-mediated signaling and activates SOCE by depleting Ca^2+^ from the ER stores (Holowka et al., 2012). For this response, addition of C2-ceramide at the optimal dose of 32 µM also rapidly inhibits the SOCE response, with a somewhat longer halftime of ∼100 s (Fig. 4B). We observed similar inhibition with optimal doses of C6-ceramide (data not shown). These results indicate that the effects of these short chain ceramides are relatively rapid, consistent with their directly affecting plasma membrane structure.

Because thapsigargin-stimulated SOCE by-passes FcεRI-mediated signaling, it appears that this inhibition by short chain ceramides occurs both at initially stimulated tyrosine phosphorylation and at a downstream step. We considered other steps that may depend functionally on an Lo-like membrane environment, including our earlier results with cholesterol depletion suggesting that engagement of the SOCE channel, Orai1, occurs in these domains (Calloway et al., 2011). To investigate this possibility further, we modified our detergent-based assay that distinguishes proteins that prefer Lo-like environments (detergent insoluble) from those that do not (detergent soluble). In particular, we demonstrated previously that very low concentrations of the non-ionic detergent, Triton X-100 (TX-100; ≤0.05% w/v), solubilize uncrosslinked IgE/FcεRI complexes on RBL cells, but are insufficient to solubilize these complexes following their specific crosslinking by a high avidity multivalent ligand (Field et al., 1997). Under similar conditions, we find that other Lo-preferring proteins, including YFP-GPI and LAT-mEGFP, are >70% resistant to solubilization by TX-100, whereas Ld-preferring proteins, such as the transmembrane protein YFP-GT46 (Kenworthy et al., 2004), are almost completely solubilized. Our previous assay was carried out on suspended cells and required separation of cell lysates on sucrose gradients. We modified our assay for adherent cells such that detergent resistant proteins remain associated with the adherent cell body and detergent soluble proteins (e.g. YFP-GT46) are washed away (Fig. S1; D.H., unpublished).

**Fig. 5.**
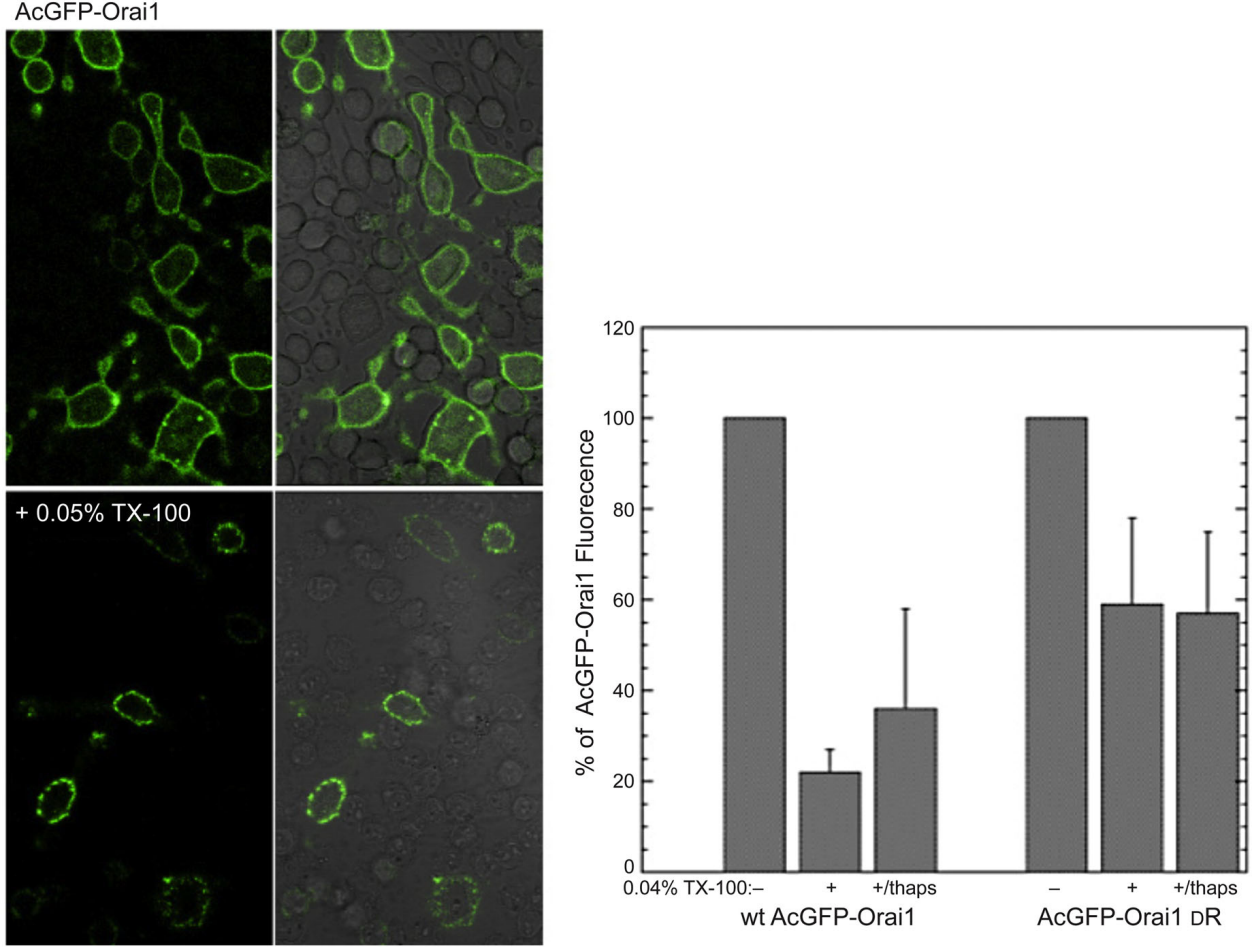
**The Ca^2+^ entry channel Orai1 is resistant to solubization by low concentrations of Triton X-100.** RBL cells were transfected with monomeric AcGFP-Orai1 (upper left panels), then treated with 0.05% TX-100 in 4°C, fixed in paraformaldehyde, and imaged by confocal microscopy (lower left). Right panels show overlay of fluorescence with brightfield images. Plot shows a summary of detergent resistance to solubilization by 0.04% Triton X-100 at 4°C for wild-type AcGFP-Orai1 and the mutant of this protein that has the basic residues 28-33 excised (AcGFP-Orai1 ΔR, Calloway et al., 2011).

Orai1 is known to be the transmembrane channel responsible for store-operated Ca^2+^ entry into the cells. If Orai1 function depends on an Lo-like membrane environment, disrupted by short-chain ceramides, then we may be able to detect resistance to detergent solubilization. As shown in Fig. 5, left panels, AcGFP-Orai1 expressed in RBL cells shows resistance to solubilization by 0.05% TX-100: some AcGFP-Orai1 is still observed at the plasma membrane in many of the transfected cells, in contrast to almost complete solubilization of transmembrane YFP-GT46 under the same conditions (Fig. S1). We quantified resistance to solubilization in 0.04% TX-100 for multiple experiments, for AcGFP-Orai1 and also for an Orai1 mutant with six positively charged amino acids in its N-terminal cytoplasmic segment deleted (Calloway et al., 2011). As summarized in Fig. 5, right panel, AcGFP-Orai1 and the reduced positive charge mutant exhibited ∼20-30% and ∼60% detergent resistance, respectively, with or without stimulation by thapsigargin. These results provide evidence that the store-operated Ca^2+^ entry channel, Orai1, is an Lo-preferring protein, and they are consistent with the possibility that the inhibition of SOCE by short chain ceramides is due to disruption of ordered lipids in the plasma membrane at this stage of the process, in addition to the disruptive effects on tyrosine phosphorylation necessary for initial stages of FcεRI-mediated signaling.

To consider the generality of our results, we evaluated the T cell receptor (TCR) for antigen. TCR is structurally similar to FcεRI: it is a multisubunit transmembrane protein complex including subunits with ITAM sequences that are tyrosine phosphorylated by Src-family kinases upon clustering of this receptor, normally via binding to peptide-containing MHC complexes on an opposing antigen-presenting cell (Zinkernagel and Hengartner, 2001). Consequent downstream signaling is also similar: tyrosine phosphorylation of TCR by Lck promotes assembly of multiple proteins, leading to generation of IP_3_ and activation of Ca^2+^ mobilization – including SOCE – that promotes exocytosis and participates in transcriptional activation of cytokine genes (Hogan et al., 2003). Partially because TCR signaling typically occurs at a cell-cell interface, a role for ordered lipids in this process remains more controversial than for FcεRI signaling. For example, a case has been made for a protein-based mechanism of TCR signaling that does not depend on lipid-mediated segregation of signaling components (James and Vale, 2012).

**Fig. 6.**
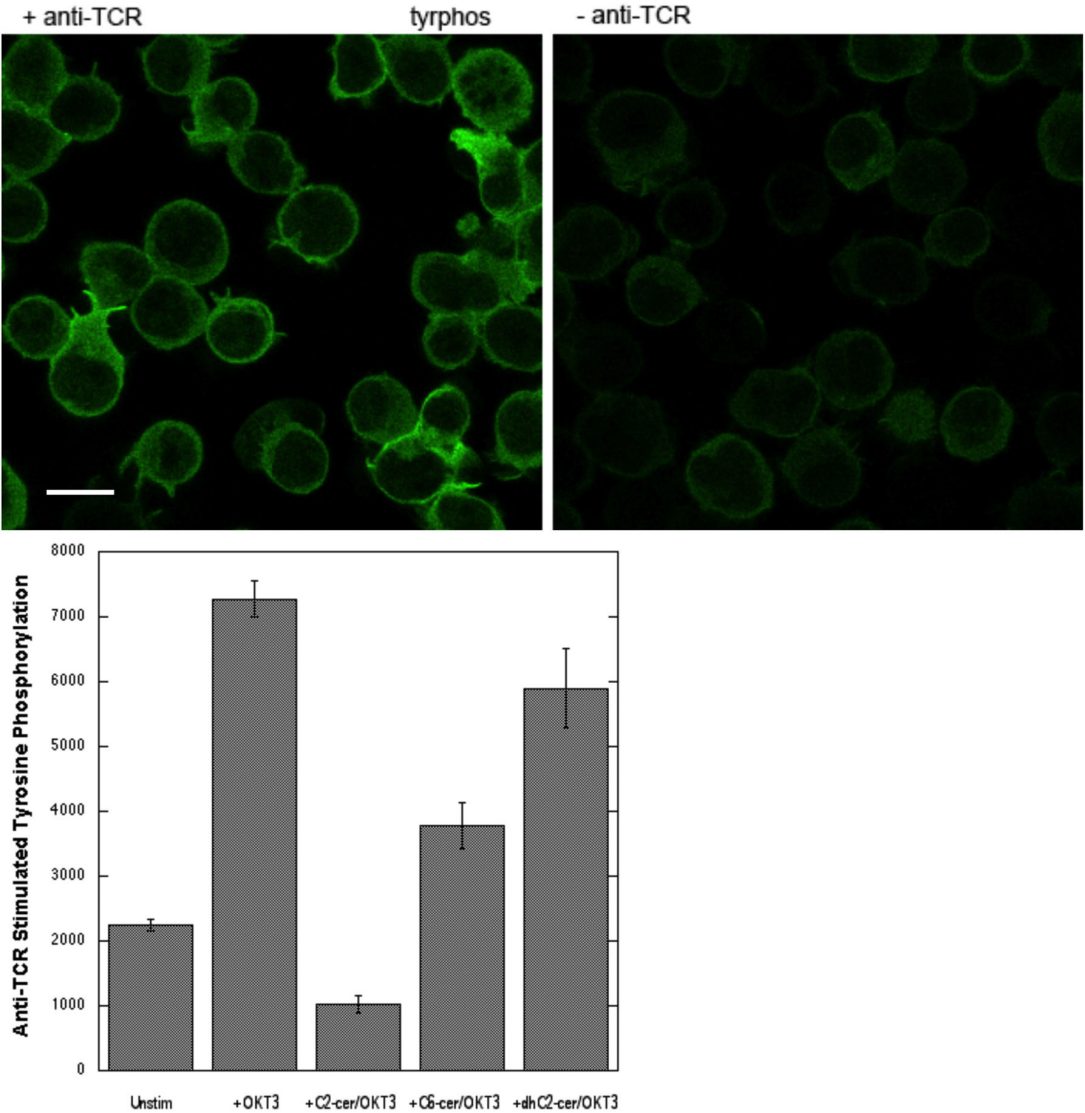
**Anti-TCR stimulated tyrosine phosphorylation in Jurkat T cells is inhibited by short chain ceramides.** Top: representative fields +/− anti-TCR OKT3. Scale bar: 5 μm. Bottom: Results from a single experiment in which anti-phospho-tyrosine fluorescence per cell is quantified by confocal imaging in more than 100 cells for each condition. Cells were stimulated for 5 min at 37°C in the absence or presence of 16 μM C6-ceramide, 32 μM C2-ceramide or 32 μM C2-dihydroceramide in BSS buffer with 1 mg/ml BSA. Cells were then processed and analyzed as for the RBL cells in Fig. 3.

**Fig. 7.**
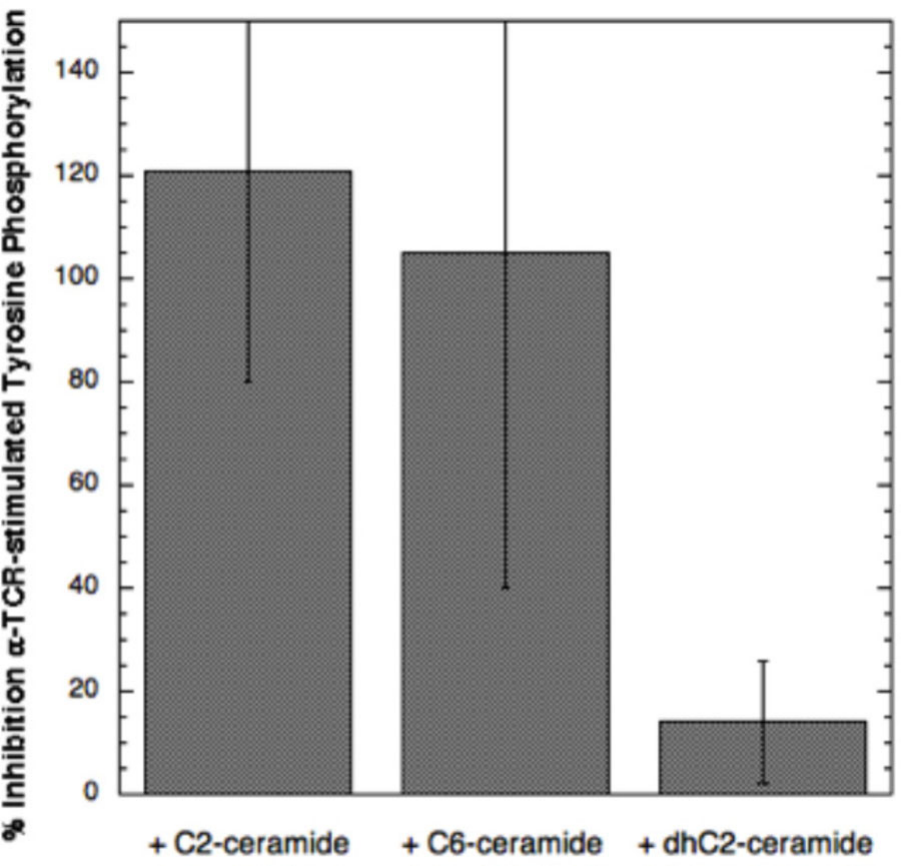
**C2- and C6-, but not C2-dihydroceramide, inhibit anti-TCR-stimulated tyrosine phosphorylation.** Values for inhibition percentage averaged over three separate experiments similar to that in Fig. 6. Error bars are ±s.d.

To test whether short chain ceramide-mediated perturbation of membrane structure affects TCR signaling similarly to that for FcεRI, we initially examined tyrosine phosphorylation stimulated by OKT3, a mAb specific for TCR (Kung et al., 1979). As shown in Fig. 6, top panels, stimulation by OKT3 causes a substantial increase in tyrosine phosphorylation of Jurkat cells, a commonly used human T cell leukemia cell line (Abraham and Weiss, 2004). We found that preincubation with C2-ceramide prior to stimulation by OKT3 reduces the basal level of phosphotyrosine in these cells and completely inhibits the stimulated response (Fig. 6, lower panel). In the experiment shown in Fig. 6, pre-addition of C6-ceramide causes partial inhibition of the tyrosine phosphorylation stimulated by OKT3 (∼40%), but, in multiple experiments, this inhibition was ∼100% on average, as summarized in Fig. 7. We also tested inhibition of stimulated tyrosine phosphorylation by C2-dihydroceramide, an analogue that, unlike C2- and C6- ceramides, has little or no biological activity (Hannun and Obeid, 2002; Gidwani et al., 2003). In our experiments, C2-dihydroceramide inhibited the anti-TCR stimulated response by <10% on average in three independent experiments (Fig. 7).

**Fig. 8.**
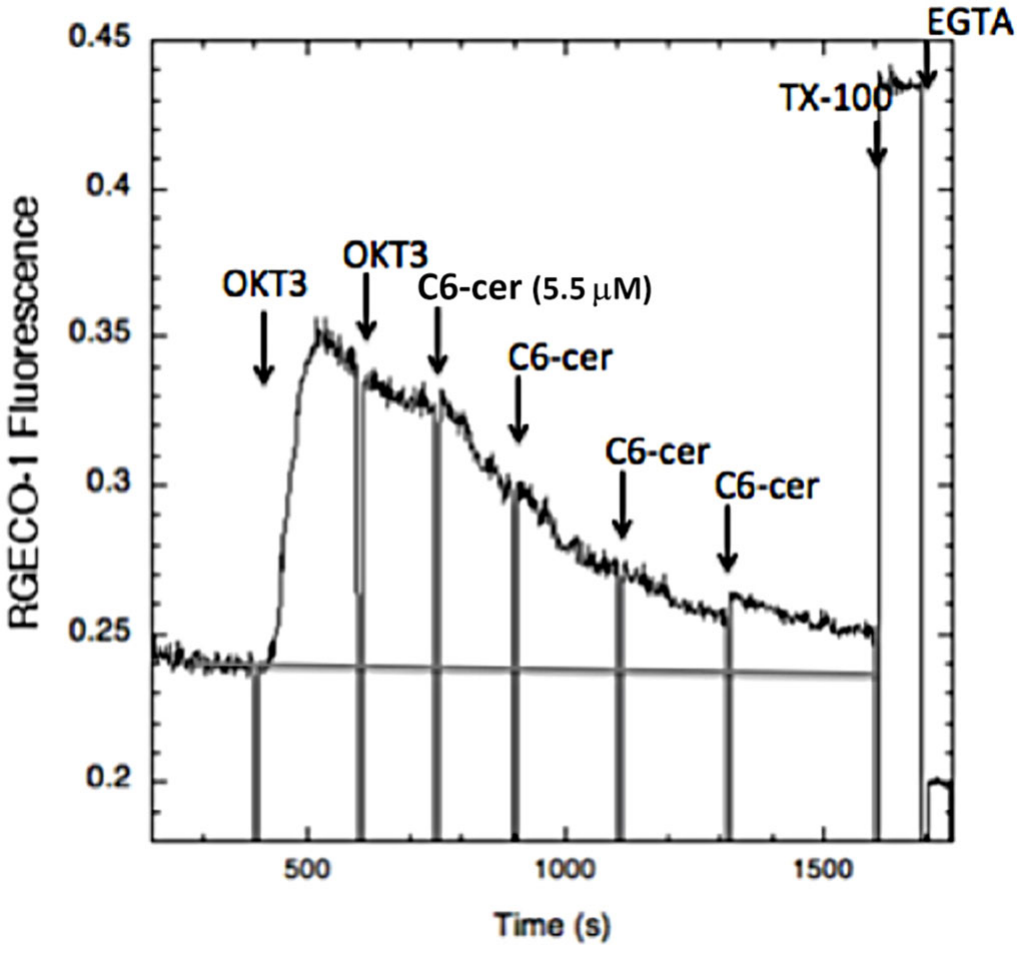
**C6-ceramide inhibits the Ca^2+^ response to TCR crosslinking by 0.2 µg/ml mAb OKT3 in Jurkat T cells expressing the cytoplasmic Ca^2+^ reporter RGECO-1.** Sequential additions of 5.5 µM C6-ceramide to a total of 22 µM in buffer with 1 mg/ml BSA cause a net inhibition of 75% in this representative experiment.

**Fig. 9.**
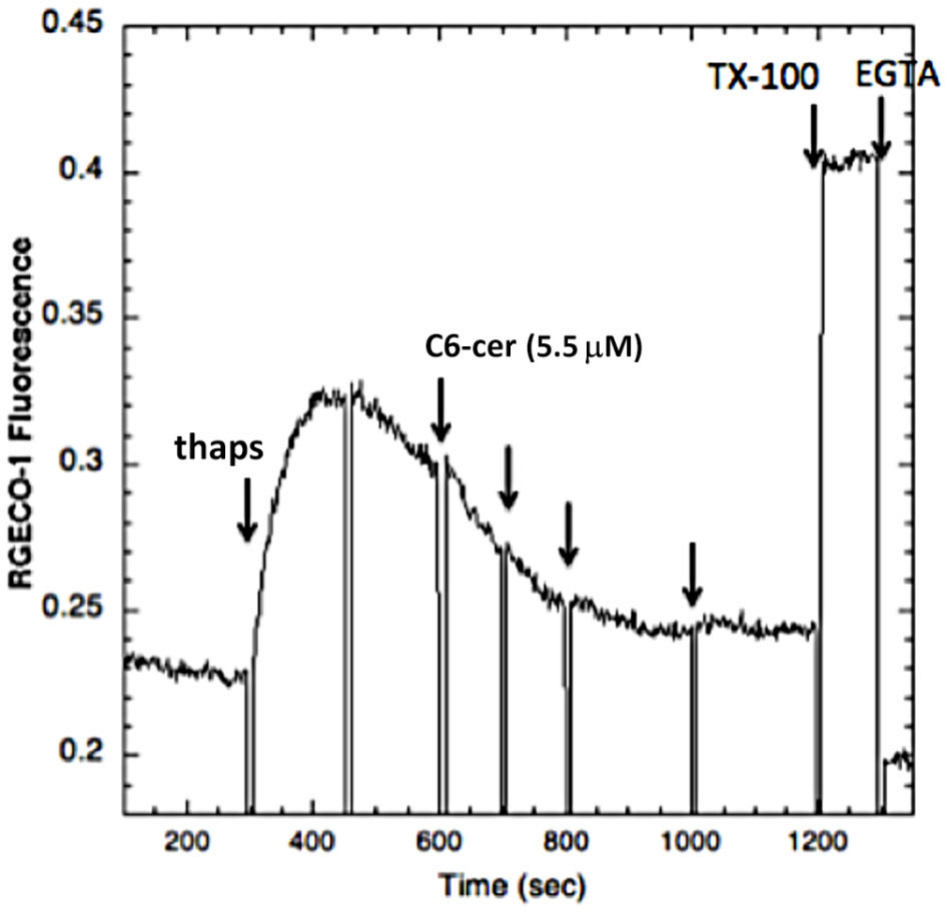
**C6-ceramide inhibits the** **Ca^2+^ response to 200 nM thapsigargin in Jurkat T cells expressing the cytoplasmic Ca^2+^ reporter RGECO-1.** Sequential additions of 5.5 µM C6-ceramide to a total of 22 μM in buffer with 1 mg/ml BSA cause a net inhibition of 78% in this representative experiment.

As a further comparison, we investigated the effects of short chain ceramides on Ca^2+^ mobilization stimulated by TCR clustering in the Jurkat T cells. We found that Jurkat T cells are more sensitive to lysis by C2-ceramide than the RBL cells, such that addition of a concentration of C2-ceramide that is optimal for inhibition of Ca^2+^ mobilization in RBL cells (32 µM) causes leakage of the Ca^2+^ indicator, R-geco1, from the T cells (data not shown). However, inhibition of Ca^2+^ mobilization in T cells by C6-ceramide is readily detected. As shown in Fig. 8, stimulation by OKT3 is progressively inhibited by increasing doses of C6-ceramide added in aliquots of 5.5 µM, with a maximal inhibition of ∼75% achieved at a final concentration of 22 µM. Similar net inhibition is observed when C6-ceramide is added prior to stimulation by OKT3 (Fig. S2). As with the RBL mast cells, stimulation of Ca^2+^ mobilization by thapsigargin is also inhibited by C6-ceramide up to a maximum of ∼78% with 22 µM (Fig. 9).

## DISCUSSION

Short chain ceramides (e.g. C2 and C6) have biological effects as well as effects on phase behavior of model membranes that differ from those of long chain ceramides (e.g. C16 and C18) which are the primary hydrolysis products of sphingomyelin. The present study related these biological and physical effects to characterize more fully the inhibition of short chain ceramides on signaling events in RBL mast cells and Jurkat T cells. We show that the earliest signaling events in both FcεRI (mast cell) and TCR (T cell) signaling, antigen-stimulated tyrosine phosphorylation, are similarly inhibited in both cell types. We further show that these short chain ceramides disrupt the Lo/Ld phase separation that is observed in GPMVs derived from the RBL mast cells, suggesting a direct effect on plasma membrane structure. These findings, together with other accumulated evidence that ordered lipid regions within the plasma membrane play a targeting role in FcεRI signaling, argue for the importance of lipid phase-like properties in signaling mediated by both these types of antigen receptors.

We previously showed that short chain ceramides, C2- and C6-, increase the fluidity of GPMVs, as monitored with the lipid analogue, diphenylhexatrienyl propanoyl phosphatidyl choline [DPH-PC; (Gidwani et al., 2003)]. More recently, we found that these ceramides also increase the fluidity of the plasma membrane of intact cells monitored by trimethylammonium-diphenylhexatriene, which inserts into its outer leaflet but cannot redistribute to other membranes because of its positively charged head group [Kuhry et al. (1983); K.T., D.H., B.B., unpublished]. These results are consistent with our findings that C2-ceramide inhibits the Lo/Ld phase separation in GPMVs that normally occurs below 15-25°C (Fig. 2). Together, these results support the view that short chain ceramides inhibit functional responses in both mast cells and T cells by interfering with the packing of the Lo-preferring lipids in the plasma membrane.

Our previous studies provided strong evidence that an Lo-like environment in the plasma membrane is important for stimulated tyrosine phosphorylation mediated by membrane-anchored Lyn kinase coupling with antigen-crosslinked IgE/FcεRI complexes (Holowka et al., 2005). The phosphorylated ITAM sequences within FcεRI subunits recruit and activate Syk tyrosine kinase, which in turn phosphorylate multiple proteins as the proximal signaling complexes assemble leading to Ca^2+^ mobilization and downstream cellular responses. Thus, an important test is sensitivity of the initial tyrosine phosphorylation events to inhibition by short chain ceramides, which disrupt the Lo phase. As shown for FcεRI in RBL mast cells, both C2-ceramide and C6-ceramide effectively inhibit this antigen-stimulated tyrosine phosphorylation (Fig. 3). Similarly, the earliest steps in signaling by TCR are the phosphorylation of ITAM tyrosines in multiple subunits by the Src family kinase, Lck, analogous to Lyn (Barber et al., 1989). Syk-family tyrosine kinase, ZAP70, is then recruited and activated to phosphorylate enzymes and scaffolding proteins leading to Ca^2+^ mobilization and downstream processes (Chan et al., 1992). As shown in Fig. 6, and summarized for multiple experiments in Fig. 7, clustering of TCR by the mAb OKT3 stimulates tyrosine phosphorylation that is prevented by the short chain ceramides C2- and C6-ceramide. In contrast to these effects, the short chain analogue C2-dihydroceramide causes only a small amount of inhibition of tyrosine phosphorylation, on average, consistent with its minimal of effect on lipid order as measured by fluorescence anisotropy of DPH-PC in GPMVs (Gidwani et al., 2003).

Earlier studies demonstrated the dependence of Ca^2+^ mobilization activated by clustering of FcεRI or TCR on stimulated tyrosine phosphorylation (Rivera and Gilfillan, 2006; Weiss et al., 1991). As the initiating phosphorylation events for both of these receptors is sensitive to inhibition by the short chain ceramides, the stimulated mobilization of Ca^2+^ is expected to be similarly sensitive, and these are our experimental results (Figs 4, 8 and 9). Added acutely, this inhibition by short chain ceramides is relatively rapid for both receptors; the dose-dependence is consistent with their effects on Lo/Ld segregation in GPMVs (Fig. 2). Also consistent, we previously showed that addition of C2- and C6-ceramide increases the distance between Lo-preferring proteins in RBL cells as measured by fluorescence resonance energy transfer (Gidwani et al., 2003), and we recently repeated these results (D.H., unpublished).

We further found that SOCE activated by thapsigargin is also sensitive to inhibition by these short chain ceramides. In this case, receptor-mediated tyrosine phosphorylation is bypassed, and STIM1 in the ER membrane coupling with Orai1 to open this Ca^2+^ channel in the plasma membrane is activated by passive depletion of Ca^2+^ from the ER stores. The only plasma membrane protein that is known to play a critical role in this process is Orai1, and we determined that this tetraspan protein exhibits detergent resistance consistent with a preference for an Lo environment. Thus, our results indicate that not only do clustered FcεRI and TCR share a preference for an Lo-like region in the plasma membrane, but the principal Ca^2+^ channel in these cells, Orai1, also functions optimally in this environment.

In summary, we have shown that short chain ceramides inhibit the earliest steps in signaling initiated by both FcεRI and TCR, as well as the downstream signaling process of Ca^2+^ mobilization in each cell type. Our results further demonstrate that these short chain ceramides are useful tools for assessing the role of ordered membrane lipids in cell signaling.

## MATERIALS AND METHODS

### Chemicals and reagents

Thapsigargin, phorbol 12,13-dibutyrate, C2-ceramide and C6-ceramide and C2-dihydroceramide were purchased from Sigma-Aldrich. FuGENE HD was from Promega (Madison, USA), and *Trans*IT/Jurkat was from Mirus Corporation (Madison, USA). mAb OKT3 and mAb 4G10 were from Thermo Fisher Scientific and A488-conjugated goat anti-mouse γ2b was from Invitrogen. The genetically encoded Ca^2+^ indicator RGECO-1 (Zhao et al., 2011) was purchased from Addgene (plasmid #32444; Cambridge, USA). A488-CTxB was from Invitrogen and TX-100 Surfact-Amps was from Thermo Fisher Scientific.

### Cells and transfection

RBL-2H3 mast cells were maintained in monolayer culture through weekly passage as described previously (Gosse et al., 2005). For stimulation, cells were sensitized with 1 μg/ml anti-DNP IgE (Gosse et al., 2005) for 2-24 h. For transfection, cells were sparsely plated (1-3×10^5^/ml) in six-well plates for fluorimetry experiments, or on #1.5 coverslips or in 35 mm cover slip dishes (MatTek Corporation, Ashland, USA) for confocal imaging. After overnight culture, cells were transfected using 2.5 μg DNA and 10 μl FuGENE HD (Promega) in 1 ml OptiMEM per well for 3-4 h in the presence of 1 ng/ml phorbol 12,13-dibutyrate to enhance DNA uptake for RBL cells (Gosse et al., 2005). Samples were then washed into full media and cultured for 16-24 h to allow for protein expression.

Jurkat T cells were maintained in suspension culture in RPMI medium with 10% fetal bovine serum and 1 μg/ml gentamicin. These cells were transfected using *Trans*IT/Jurkat from Mirus Corporation for 16-24 h per manufacturer's recommendations.

Cells were then washed in buffered salt solution (BSS: 135 mM NaCl, 5 mM KCl, 1 mM MgCl_2_, 1.8 mM CaCl_2_, 5.6 mM glucose, 20 mM HEPES, pH 7.4) with 1 mg/ml bovine serum albumin for experiments with short chain ceramides.

### Confocal microscopy

GPMVs were elicited from RBL cells as previously described (Sengupta et al., 2008) and allowed to settle overnight at 4°C. After concentrating by removal of ∼90% of the supernatant, the GPMVs were labeled with 5 μg/ml A488-CTxB, treated or not with 32 µM C2-ceramide for 10 min at 25°C, then cooled on ice prior to imaging by confocal fluorescence microscopy and scoring for phase separation at an equatorial plane.

For quantification of tyrosine phosphorylation, RBL cells plated overnight at a subconfluent density of 0.5×10^6^ cells/ml in 35 mm coverslip dishes were sensitized with anti-DNP IgE, then washed into BSS and stimulated or not at 37°C for 5 min in the presence or absence of short chain ceramides as specified, then fixed in 4% para-formaldehyde with 0.1% glutaraldehyde and quenched with 10 mg/ml BSA in PBS with 0.01% sodium azide. Cells were then labeled with 5 μg/ml anti-phosphotyrosine in 0.1% TX-100, followed by A488-anti-mouse γ2b. Confocal imaging was performed using a Zeiss (Darmstadt, Germany) LSM 710 inverted confocal microscope with a 63× Oil Plan-Apochromat lens and analyzed by ImageJ. Typically, fluorescence intensity from 135×135 μm equatorial non-saturated confocal images containing 15 to 35 cells was integrated, and the average fluorescence intensity per cell was determined. This fluorescence intensity per cell was averaged for at least four such images for each sample and tabulated ±s.d. for at least three independent experiments. Jurkat T cells were plated onto poly-D-lysine-coated coverslip dishes, then incubated at 37°C for 5 min in the presence or absence of short chain ceramides and stimulated or not with 0.5 μg/ml OKT3 for 5 min at 37°C before fixing and labeling with anti-phosphotyrosine as above, with similar quantitative analysis of equatorial confocal images as for the RBL cells. Inhibition percentage was calculated as:

[(+antigen/OKT3+ceramide)-unstimulated/ (+antigen/OKT3-unstimulated)] ×100.

Detergent resistance of AcGFP-Orai1 and other fluorescent proteins, including mYFP-GT46, was quantified in the presence and absence of 0.04% TX-100 in BSS, incubated for 10 min at 4°C, then fixed similarly to anti-phosphotyrosine imaging: fluorescence intensity from 135×135 μm equatorial non-saturated confocal images containing 15 to 35 cells per image was integrated with ImageJ software, and the average fluorescence intensity per cell was determined in four separate experiments with five images per sample (300-700 cells for each).

### Fluorescence spectroscopy measurements

Cytoplasmic Ca^2+^ levels were measured using an SLM 8100C steady-state fluorimeter (SLM Instruments, Urbana, USA). RBL cells previously transfected with R-geco1 and sensitized with anti-DNP IgE were harvested using PBS/EDTA and resuspended in BSS. Cells were stirred in an acrylic cuvette at 37°C, and the time course of R-geco1 fluorescence (ex 560, em 580 nm LP) was monitored. Inhibition of SOCE was determined as percentage decrease in the sustained phase of the Ca^2+^ response stimulated by antigen (DNP-BSA) or thapsigargin following addition of C2- or C6-ceramide by linear extrapolation of the Ca^2+^ response from the time of addition of the inhibitor. For Jurkat T cells Similar Ca^2+^ responses were stimulated by OKT3, anti-TCR, or thapsigargin, and inhibition due to acute addition of short chain ceramides was determined in a similar manner.

## Supplementary Material

Supplementary information
