# Supplementary material for: Short chain ceramides disrupt immunoreceptor signaling by inhibiting segregation of Lo from Ld Plasma membrane components
Source: Biol Open. 2018 Aug 10;7(9):bio034702. doi: 10.1242/bio.034702 (PMC6176950; doi:10.1242/bio.034702)
Supplement: Supplementary information [file biolopen-7-034702-s1.pdf]

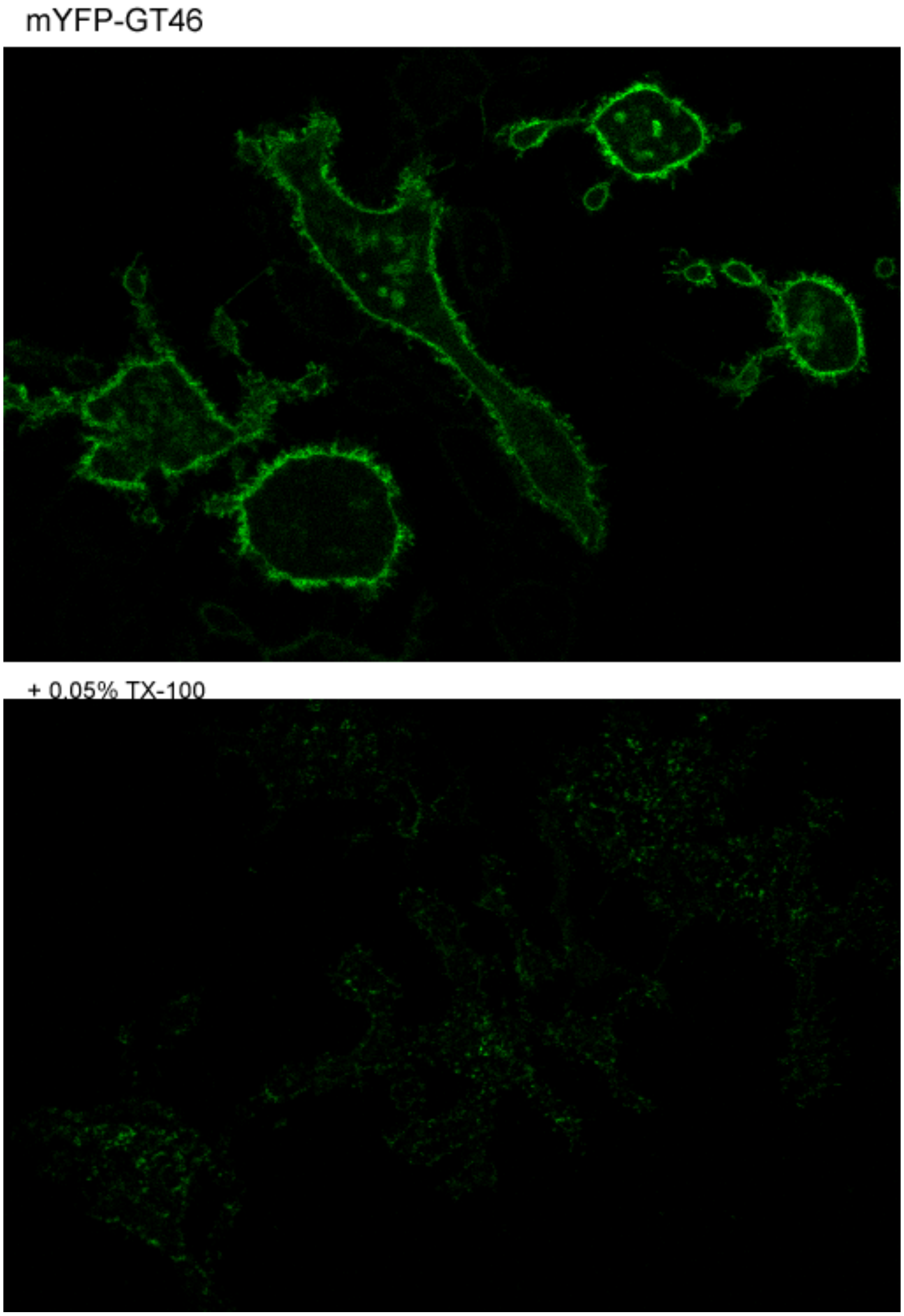

Figure S1. RBL 2H3 cells were transfected with mYFP-GT46 (Kenworthy et al., 2003), then incubated in BSS with or without 0.05% TX-100 (w/v) at 4°C and then fixed for confocal imaging. Quantification of confocal equatorial images similar to above indicated loss of greater than 95% mYFP fluorescence due to 0.04%TX-100 for >100 cells.

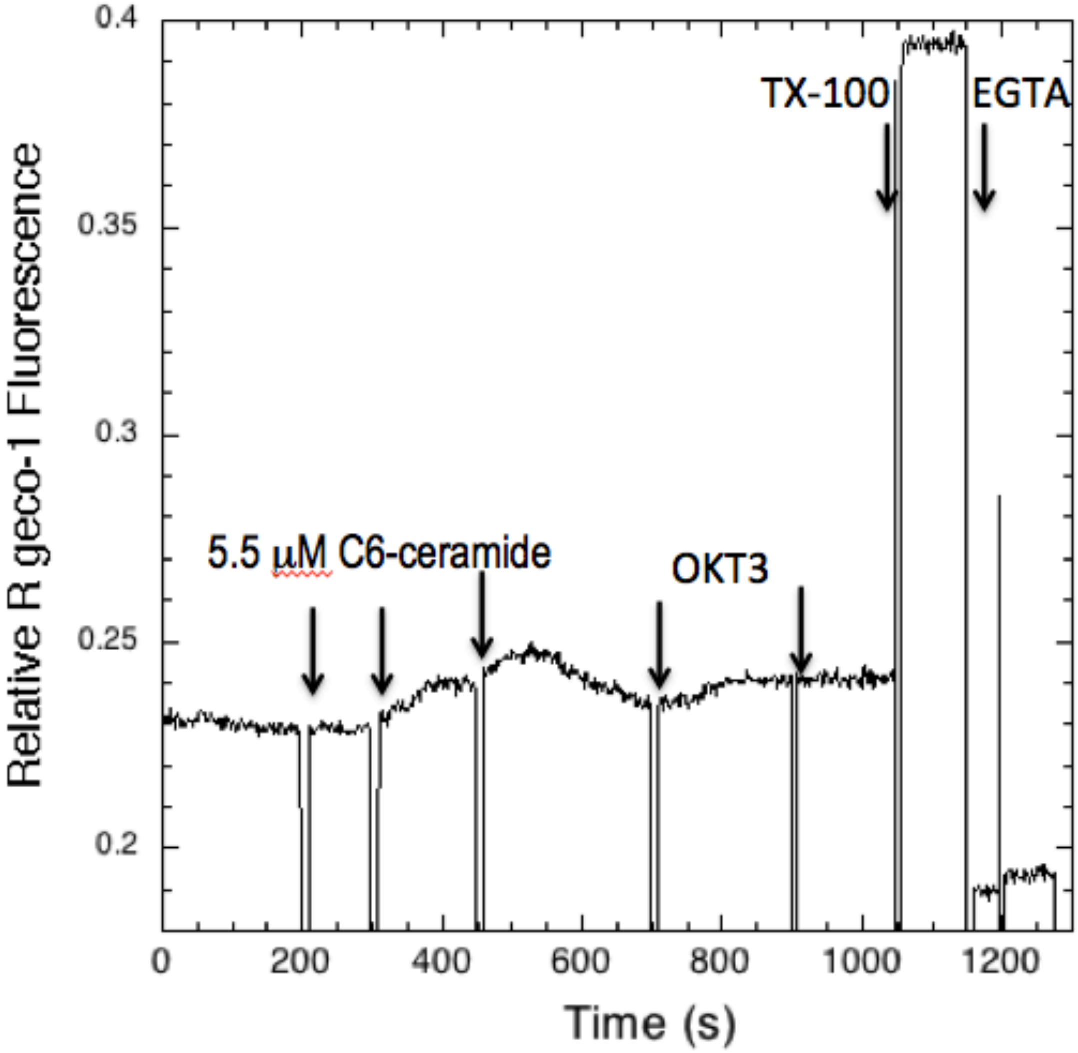

Figure S2. C6-ceramide, added at a final concentration of 16.5 μM prior to stimulation by 0.15 μg/ml OKT3 largely inhibits the Ca^{2+} response to TCR in Jurkat cells.

Biology Open • Supplementary information
